# Resampling methods to reduce the selection bias in genetic effect estimation in genome-wide scans

**DOI:** 10.1186/1471-2156-6-S1-S24

**Published:** 2005-12-30

**Authors:** Long Yang Wu, Sophia SF Lee, Haijiang Steven Shi, Lei Sun, Shelley B Bull

**Affiliations:** 1 Samuel Lunenfeld Research Institute, Mount Sinai Hospital, 600 University Avenue, Toronto, Ontario, Canada M5G 1X5; 2 Department of Public Health Sciences, University of Toronto, 12 Queen's Park Crescent West, Toronto, Ontario, Canada M5S 1A8; 3 Hospital for Sick Children, 555 University Avenue, Toronto, Ontario, Canada M5G 1X8

## Abstract

Using the simulated data of Problem 2 for Genetic Analysis Workshop 14 (GAW14), we investigated the ability of three bootstrap-based resampling estimators (a shrinkage, an out-of-sample, and a weighted estimator) to reduce the selection bias for genetic effect estimation in genome-wide linkage scans. For the given marker density in the preliminary genome scans (7 cM for microsatellite and 3 cM for SNP), we found that the two sets of markers produce comparable results in terms of power to detect linkage, localization accuracy, and magnitude of test statistic at the peak location. At the locations detected in the scan, application of the three bootstrap-based estimators substantially reduced the upward selection bias in genetic effect estimation for both true and false positives. The relative effectiveness of the estimators depended on the true genetic effect size and the inherent power to detect it. The shrinkage estimator is recommended when the power to detect the disease locus is low. Otherwise, the weighted estimator is recommended.

## Background

After a genetic marker or candidate gene has been identified from a genome-wide scan as a putative disease susceptibility locus, it is of interest to estimate the associated genetic effect on the related phenotype. However, locus-specific effect estimates are subject to upward selection bias because of stringent test criteria adopted in genome-wide scans. Göring et al. [1] formally raised this issue and argued that reliable locus-specific parameter estimates can only be obtained in an independent sample. Sun and Bull [2] proposed three resampling-based estimators that can be applied to the original sample at the location where the maximum test statistic exceeds a genome-wide significance criterion. They demonstrated effective bias reduction in analytic and simulation studies of a homogenous population with a single disease gene. In their simulation studies, they compared a catalog of resampling methods, including cross-validation and bootstrapping, and their results suggested that bootstrap methods perform best in terms of smaller mean squared error. Therefore, we focused on the bootstrap method in the current study.

The simulated data of Problem 2 for Genetic Analysis Workshop 14 (GAW14) provided a microsatellite marker map of 416 markers with a resolution of 7 cM and a denser single-nucleotide polymorphism (SNP) marker map of 917 markers with 3-cM density. The disease expression was under the influence of multiple genes in a complex manner. We compared performance of the two maps in multipoint linkage analysis in terms of power and localization accuracy. The main objective of this study was to further investigate the effectiveness of bootstrap resampling methods in reducing the bias of genetic effect estimates in genome-wide linkage scans. The new methods, were applied to both the microsatellite and SNP data for selected replicates. With the knowledge of the answers to the simulated data, we were able to investigate the performance of the new methods under stratification of true and false positives.

## Methods

To evaluate the power to detect linkage, we conducted multipoint analyses in all the 100 replicates using ALLEGRO [3]. There were four populations Aipotu (AI), Danacaa (DA), Karangar (KA), and New York (NY) in each replicate. AI, DA, and KA included only nuclear families, while NY had multigeneration extended pedigrees. Because some of the large NY families (size > 25 bits) required too much execution time to complete the analysis in a reasonable time, the NY population was excluded.

We adopted the exponential allele-sharing model of Kong and Cox [4] and used *Spair *as the scoring function for affected relatives. The genetic effect was measured by *δ *the excess identity-by-descent (IBD) allele-sharing parameter in this model. The genome-wide significance criterion was set to 2.2 × 10^-5 ^[5], corresponding to a *Zlr *value of 4.09, where *Zlr *is the test statistic for linkage in the exponential model. In each replicate of the three populations, we identified all loci that met the significance criterion.

We implemented a simple bootstrapping method in this study. Suppose that the original dataset has *n *families; we repeatedly drew random samples of size *n *with replacement from it. In each bootstrap replication *b *(*b *= 1, ..., *B*), the selected families constitute the detection sample, and the remaining families (out-of-sample families) comprise the estimation sample, thus providing independence within each of the *B *resampling replications. To reduce the upward selection bias in the genetic effect estimates of δ we implemented three bootstrap-based estimators [2]: a shrinkage estimator defined by , an out-of-sample estimator , and a weighted estimator , where *ω *= 0.632 was analogous to Efron's 0.632 estimator [6].

We first obtained the naïve estimate, , at location *m*_*D*_, where the maximum test statistic exceeded the significance criterion in the original data. Note that the location *m*_*D *_was the overall gene localization and the three bootstrap-based estimators were then applied only to genetic effect estimation at this location. The shrinkage estimator was constructed by reducing the naïve estimate  by a shrinkage factor of , which was constructed by taking the average of the difference between  and  over B* bootstrap replications, with B* ≤ *B*, where B* is the number of replications with significant results. In bootstrap replication *b*,  is the genetic effect estimate at location  with the maximum significant genome-wide test statistic in the detection sample;  is the genetic effect estimate at the same location  in the estimation sample. Note that  could be different from *m*_*D*_. The out-of-sample estimator was the average of  at location  in the estimation sample over B* bootstrap replications. It resembles the estimate that would have been obtained in an independent sample. The weighted estimator combined  and  with the weight of *ω*. The weight was chosen to be 0.632, which was derived from a distance argument based on the fact that bootstrap samples are supported by about 0.632*n *of the original families [6,7]. Note that the weighted estimator can also be written as . Therefore, it can be considered as a variant of shrinkage estimator, with the amount of shrinkage depending on *ω *and . Although an adaptive choice of the weight is attractive, as in the 0.632+ method [7], time constraints precluded its inclusion in this study.

Bias reduction of the three estimators was compared according to whether the localization was a true or false positive. We classified significant findings in the 100 replicates into true or false positives, according to the answers (disease loci D1 and D2 on chromosomes 1 and 3 for the AI, KA, and DA populations, and disease loci D3 and D4 on chromosomes 5 and 9 for AI and KA). A true positive was defined if the detection was within 10 cM of the true disease gene location. The true genetic effects were estimated by averaging corresponding estimates from all 100 replicates.

## Results and discussion

Averaging over all 100 replicates, the genome scans based on microsatellite markers at 7-cM density yielded similar performance in power and in accuracy of location estimates to those based on SNP markers at 3-cM density (Table 1). The power to detect disease gene loci varied among populations (Table 1), and the DA population generally had the highest power among the three populations.

**Figure 1:**
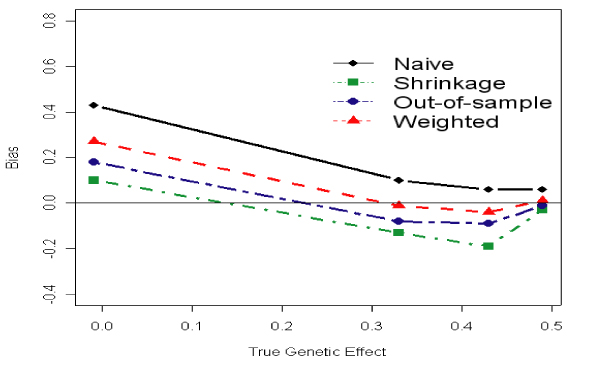
Bias comparisons of the naïve estimate and the three resampling-based estimates for microsatellite markers.

For the microsatellite marker analysis, we used replicates 1 and 35 to illustrate the application of the three bootstrap-based estimators (Table 2). Replicate 1 was used for our initial genome scan. Replicate 35 was chosen because it contained an unambiguous false positive for the DA population on chromosome 6, after the chromosome containing the locus with highest test statistic (i.e., a true positive) was removed. We confirmed that the naïve estimates overestimated the true genetic effects. In this example, the most severe overestimation occurred at the false positive location. Figure 1 depicts the biases of naïve and bootstrap-based estimates at various levels of true genetic effect. The bootstrap-based estimates were less biased than the naïve estimate for both true and false positives. When the true genetic effect was relatively large and power was high, such as the location at 169.97 cM on chromosome 1 of DA population (Table 2), the three estimators gave roughly the same genetic effect estimate and had low bias. When the true genetic effect was moderate, such as the significant loci on chromosome 3 of AI population and chromosomes 5 and 9 of KA population, the three estimates were different. The shrinkage estimates overcorrected, while the weighted estimates were least biased. In the case of a false positive, i.e., the significant locus at 192.09 cM on chromosome 6 of DA population, the shrinkage estimate gave the best result in terms of bias, followed by the out-of-sample estimate and the weighted estimate.

**Figure 2:**
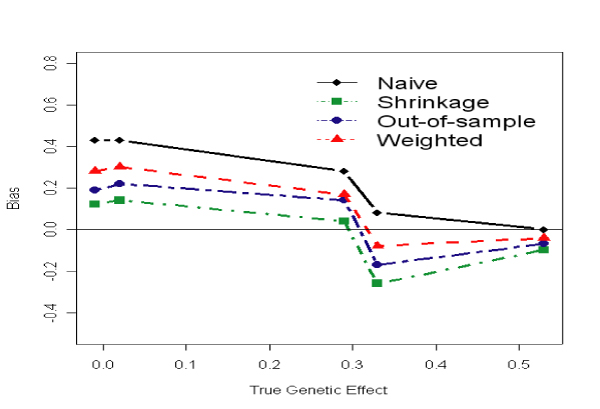
Bias comparisons of the naïve estimate and the three resampling-based estimates for SNP markers.

The bias reductions for the SNP marker analysis are presented in Table 2. We report results for replicates 1, 27, and 67. After the chromosomes with highest test statistic (true positives) were removed, we found two false positives at chromosome 9 (replicate 27) and chromosome 5 (replicate 67) for DA population. The bias reduction pattern was similar to the pattern in microsatellite markers (Figure 2). Note that selection bias in the naïve estimates was similar for microsatellite and SNP analysis, despite having twice as many markers for the latter.

The bootstrap-based estimators reduced the upward selection bias in genetic effect estimation for both microsatellite and SNP based linkage analysis. The performance of the three estimators differed according to true or false positive status. The shrinkage estimator had the smallest bias for false positives but over-corrected for the true positives. On the other hand, the weighted estimator had the smallest bias for true positives but under-corrected for the false positives. It has been shown that the bias depends on the power to detect linkage [2]. In these examples from the simulated data, we found that the shrinkage estimator had lower bias when the power was less than 20%. Otherwise, the weighted estimator provided lower bias.

In this study, our bootstrap estimators focused on genetic effect estimation for the most significant locus in a genome scan, without considering other loci that also exceeded genome-wide significance criteria. However, the underlying genetic model has multiple loci. Further research is warranted to construct a joint estimator that would simultaneously handle multiple significant loci and thereby extend bias-reduction methods to more general settings.

## Conclusion

The reliability of gene detection, the accuracy of locus-specific effect estimates, and the failure to replicate initial claims of linkage or association have emerged as major concerns in genome-wide studies. Estimation of the genetic effect for a specific locus in a genome-wide scan is subject to upward bias because of selection by strict significance criteria. This bias is most severe for locations with small genetic effect and low power. Our results indicate that, in a complex disease setting, the three bootstrap-based estimators appear to be effective in reducing the selection bias of the naïve estimator. The shrinkage estimator is recommended when the power to detect the disease loci is low. Otherwise, the weighted estimator is recommended.

## Abbreviations

GAW: Genetic Analysis Workshop

IBD: Identity by descent

SNP: Single-nucleotide polymorphism

## Authors' contributions

LYW implemented the bootstrap methods and drafted the manuscript. SSFL assisted in preparing the manuscript for publication. HSS conducted the genome-wide MS and SNP scans. LS and SBB developed the bootstrap estimators and assisted in revising the manuscript. All authors read and approved the final manuscript.

## Figures and Tables

**Table 1:** Comparison of linkage analysis results between microsatellite and SNP based genome scans

			Location estimates (mode)		Power to detect linkage		Mean test statistic	
Pop	Chr.	True location (cM)	MS (cM)	SNP (cM)	MS	SNP	MS	SNP

AI	1	168.98	169.85	167.4	4/100	7/100	4.33	4.56
	3	299.32	293.61	295.6	**22/100^a^**	**24/100**	4.79	4.77
	5	5.45	7.34	5.94	11/100	11/100	4.53	4.63
	9	5.88	4.78	5.54	10/100	9/100	4.39	4.38

KA	1	168.98	169.97	168.0	2/100	2/100	4.07	4.24
	3	299.32	293.75	295.9	**14/100**	**17/100**	4.41	4.77
	5	5.45	7.01	5.69	20/100	40/100	4.67	4.71
	9	5.88	6.80	5.96	**52/100**	**45/100**	4.95	4.92

DA	1	168.98	169.95	168.8	**82/100**	**89/100**	5.20	5.43
	3	299.32	293.62	295.7	48/100	56/100	4.89	4.77

^a ^Bold text indicates the cases in which linkage was detected in replicate 1 with genome-wide significance using either the microsatellite or the SNP markers (as reported in Table 2).

**Table 2:** Comparison of linkage analysis results and genetic effect estimates for the naïve and three bootstrap estimates using microsatellite markers and SNPs.

						Bootstrap estimate (bias)			
Replicate	Population	Chromosome	Highest peak (cM)	True genetic effect	T/F positive				

Microsatellite Markers									
1	DA	1	169.97	0.49 ± 0.10	T	0.55 (0.06)	0.50 (0.01)	0.48 (-0.01)	0.47 (-0.03)
1	AI	3	294.68	0.33 ± 0.16	T	0.43 (0.10)	0.31 (-0.01)	0.25 (-0.08)	0.19 (-0.13)
1	KA	9	2.76	0.43 ± 0.11	T	0.49 (0.06)	0.40 (-0.04)	0.34 (-0.09)	0.24 (-0.19)
35	DA	6	192.09	-0.01 ± 0.11	F	0.41 (0.42)	0.26 (0.27)	0.17 (0.18)	0.10 (0.11)

SNP Markers									
1	DA	1	168.94	0.53 ± 0.10	T	0.53 (0.00)	0.49 (-0.04)	0.46 (-0.07)	0.43 (-0.10)
1	AI	3	304.58	0.33 ± 0.11	T	0.41 (0.08)	0.25 (-0.08)	0.16 (-0.17)	0.07 (-0.26)
1	KA	3	305.81	0.29 ± 0.11	T	0.57 (0.28)	0.48 (0.19)	0.43 (0.14)	0.33 (0.04)
27	DA	9	200.12	0.02 ± 0.13	F	0.45 (0.43)	0.32 (0.30)	0.24 (0.22)	0.16 (0.14)
67	DA	5	214.33	-0.01 ± 0.11	F	0.42 (0.43)	0.27 (0.28)	0.18 (0.19)	0.11 (0.12)

*Mean ± SD over 100 replicates
